# Does the Use of an Automated Resuscitation Recorder Improve Adherence to NRP Algorithms and Code Documentation?

**DOI:** 10.3390/children11091137

**Published:** 2024-09-19

**Authors:** Sarah Nelin, Simon Karam, Elizabeth Foglia, Philip Turk, Venu Peddireddy, Jagdish Desai

**Affiliations:** 1 Department of Pediatrics, University of Mississippi Medical Center, Jackson, MS 39216, USA; 2 Division of Neonatology, Children’s Hospital of Philadelphia, Philadelphia, PA 19104, USA; 3 Clinical and Translational Research Institute, Northeast Ohio Medical University, Rootstown, OH 44272, USA; 4 Pediatrix Medical Group, Neonatology, Austin, TX 78705, USA

**Keywords:** resuscitation, neonatal resuscitation program (NRP), simulation, code documentation

## Abstract

Background: Neonatal resuscitation is guided by Neonatal Resuscitation Program (NRP) algorithms; however, human factors affect resuscitation. Video recordings demonstrate that deviations are common. Additionally, code documentation is prone to inaccuracies. Our long-term hypothesis is that the use of an automated resuscitation recorder (ARR) app will improve adherence to NRP and code documentation; the purpose of this study was to determine its feasibility. Methods: We performed a simulation-based feasibility study using simulated code events mimicking NRP scenarios. Teams used the app during resuscitation events. We collected data via an initial demographics survey, video recording, ARR-generated code summary and a post-resuscitation survey. We utilized standardized grading tools to assess NRP adherence and the accuracy of code documentation through resuscitation data point (RDP) scoring. We evaluated provider comfort with the ARR via post-resuscitation survey ordinal ratings and open-ended question text mining. Results: Summary statistics for each grading tool were computed. For NRP adherence, the median was 68% (range 60–76%). For code documentation accuracy and completeness, the median was 77.5% (range 55–90%). When ordinal ratings assessing provider comfort with the app were reviewed, 47% chose “agree” (237/500) and 36% chose “strongly agree” (180/500), with only 0.6% (3/500) answering “strongly disagree”. A word cloud compared frequencies of words from the open-ended text question. Conclusions: We demonstrated the feasibility of ARR use during neonatal resuscitation. The median scores for each grading tool were consistent with passing scores. Post-resuscitation survey data showed that participants felt comfortable with the ARR while highlighting areas for improvement. A pilot study comparing ARR with standard of care is the next step.

## 1. Introduction

Neonatal resuscitation is the most commonly performed resuscitation in hospitals, with approximately 5% of babies born at term requiring higher-level resuscitation interventions [1]. Decreasing gestational age is associated with an increasing need for resuscitative interventions, with as many as 92% of extremely preterm infants requiring some intervention in the delivery room [2]. Bajaj et al. have shown that an increased intensity of delivery room resuscitation was associated with adverse neonatal outcomes [3]. The Neonatal Resuscitation Program (NRP) is the training standard in the United States, providing guidelines for an evidence-based, timely and coordinated response [4]. The first resuscitative intervention in NRP is positive-pressure ventilation (PPV). Perlman et al. showed that ineffective or improper ventilatory support leads to continued neonatal depression, indicating that inappropriately following NRP algorithms leads to the need for interventions such as chest compressions or epinephrine, and subsequent poor outcomes [5]. Video recordings of resuscitation have been used to monitor performance and adherence to NRP guidelines in several studies, which found that up to 54% of resuscitations have some form of deviation from the NRP guidelines, with one study finding that only 21% of interventions were performed according to guidelines [6,7,8].

The documentation of resuscitative efforts (code documentation) across all areas of the hospital is another aspect of resuscitations that shows significant variation and inaccuracy, with most code documentation relying on memory. Real-time code documentation is essential for improving resuscitation efforts, post-resuscitation care and family counseling. Braga et al. showed that neonatal resuscitation documentation varies significantly across institutions and highlighted the need for the standardization of code documentation [9]. In a systematic review by Avila-Alvarez et al., it was noted that code documentation was inaccurate or unsatisfactory 39–54% of the time, with specific interventions documented appropriately 13–99% of the time, and the authors noted that complex or advanced procedures were more accurately recorded than basic interventions [10]. APGAR scores are a part of code documentation unique to neonatal resuscitations, with multiple studies showing variation in APGAR scoring and inaccuracies when scored by memory and not in real time [6,11].

Both adherence to NRP guidelines and code documentation rely heavily on human factors for appropriate completion. Several studies have looked at app-based programs to improve compliance with NRP guidelines during delivery room resuscitation and have shown improved performance of NRP guidelines. Fuerch et al. showed that using a decision support tool significantly increased PPV and chest compression performance during NRP simulations, as well as increasing the number of times FiO_2_ was addressed during resuscitation [12]. While this study examined three key areas of NRP resuscitation, it did not assess team performance through a comprehensive assessment of NRP performance or assess code documentation accuracy. Dinur et al. studied the use of an audio-voice guided application showing improved adherence to and performance of NRP guidelines in resuscitation simulations, as compared to non-guided resuscitation [13]. Roitsch et al. also performed a study analyzing the effect of a tablet-based decision support tool on adherence to NRP guidelines and found improved compliance with NRP both in decision making and in performance of skills [14]. Benguigui et al. studied a digital cognitive aid in comparison to a poster cognitive aid used in neonatal resuscitation, finding improved technical scores via a standardized NRP checklist [15]. While the latter three studies are randomized, simulation-based studies evaluating NRP performance comprehensively, they do not evaluate code documentation or other aspects of neonatal delivery room care. To the best of our knowledge, app-based programs have not been studied as a means for improving code documentation in addition to NRP adherence thus far.

In summary, while NRP provides the framework for neonatal resuscitation events, video recording has shown significant deviations from the guidelines. In addition, code documentation of resuscitation is prone to inaccuracies. The addition of app-based technology may improve neonatal resuscitation events. The purpose of this study is to assess the feasibility of app-based technology in neonatal resuscitation simulations. Our long-term hypothesis is that the use of the automated resuscitation recorder (ARR) app will improve adherence to NRP algorithms and code documentation. We also hypothesize that the use of the ARR app will improve provider comfort level and confidence in resuscitation.

## 2. Materials and Methods

The ARR app was invented and developed at the University of Mississippi Medical Center (UMMC). The application has two main components: (1) event recording and (2) voice guidance. Event recording is performed via manual buttons on the device, with all inputs recorded in real-time. Voice guidance is provided by the ARR app for basic NRP interventions, including PPV, MR. SOPA corrective steps, intubation and chest compressions. The voice guidance is not intended to lead the resuscitation, but to provide timing and reminders for NRP interventions throughout the resuscitation event. The application is divided into four “pages”; please see Supplemental Figures S1–S4 for graphics of the display. Page 1 includes pre-delivery information including patient name, gestational age and estimated fetal weight (EFW). It also includes the four pre-birth questions included in NRP, as well as an equipment checklist and resuscitation team information. Page 2 is the resuscitation page, including a display of EFW, target saturations, equipment sizing and medication dosing for the infant. It also displays real-time resuscitation event documentation and APGAR scoring tables, as well as options to record vital signs, infant assessments and interventions including routine or advanced NRP resuscitation. Page 3 includes post-resuscitation information including actual infant weight, final APGAR scoring and the disposition of the infant. Page 4 includes the final code documentation, which can be printed and/or transmitted to the electronic medical record.

We performed a simulation-based feasibility study using a convenience sample size of mock code events. The study was conducted at UMMC between July 2022 and June 2023, using the neonatal simulation lab to run simulated mock code events using standardized multidisciplinary teams mimicking NRP training scenarios. The scenario described a term delivery complicated by shoulder dystocia requiring the team to provide resuscitative measures including PPV, chest compressions and epinephrine administration. Participants were recruited from a pool of neonatal nurse practitioners (NNPs), pediatric residents rotating in the NICU, respiratory therapists and delivery room/bedside nurses in the UMMC NICU. Each resuscitation team was made up of five providers to simulate a true neonatal code event, with a provider (NNP), resident, respiratory therapist and two nurses, and with each participant only involved in one mock code event. The resuscitation team oversaw the operation of the ARR app, and typically they selected the person performing the “recorder” role for this. The study endpoint was team performance in the mock code event, assessed via standardized grading tool scoring. Each simulation team was given a pre-resuscitation demographics survey and then oriented to the ARR system prior to participation in the mock code event. Each mock code was video recorded for later review. After completion of the mock code, team members were asked to complete a post-resuscitation survey to evaluate comfort and satisfaction with the ARR app. Each participant was provided a USD 20 gift card for participation after completion of the post-resuscitation survey.

We analyzed data using three separate outcome measures: (1) adherence to NRP guidelines while using the ARR app, (2) accuracy and completeness of code documentation produced by the ARR app and (3) provider comfort level while using the ARR app. Mock code observations through video recording were used to evaluate outcomes (1) and (2), while outcome measure (3) was evaluated using post-resuscitation surveys. Each video review and scoring, as described below, was completed by the same investigator to minimize variability.

For outcome (1), adherence to NRP guidelines, a standardized 25-point NRP compliance checklist was created based on performance checklists from the NRP instructor manual. The video recording from each scenario was reviewed and graded, using this checklist, as either non-compliant (0 points) or compliant (1 point), with the final score calculated for each scenario (25 points possible). This score was then converted into a percentage of highest possible score and used for analysis.

For outcome (2), code documentation data, the ARR app created a code summary report at the end of each scenario, which was reviewed using a standardized grading tool to assess the accuracy and completeness of documentation in comparison to the video recording of the mock code event. This standardized grading tool (called the resuscitation data point, or RDP) was created to include ten key data points that are essential for complete documentation in every neonatal resuscitation. Each point was graded as absent (0 points), inaccurate (1 point) or accurate (2 points), with a final score calculated for each scenario (20 points possible). This score was then converted into a percentage of highest possible score and used for analysis.

**Figure 1:**
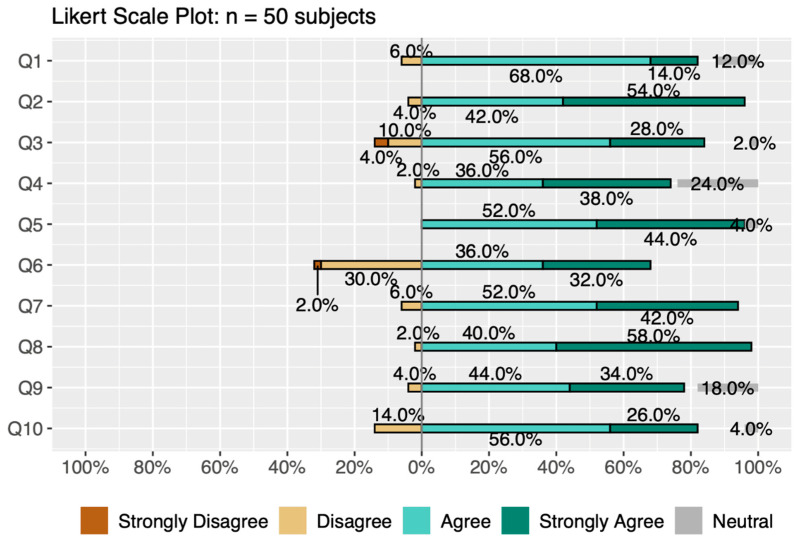
Likert scale survey data. The percentage of the total answers for each question (*n* = 50) (Q1–Q10) from strongly disagree to strongly agree are shown here. These data show that the majority of responses were either agree or strongly agree (positive assessment) for each question that was used to assess provider comfort with the ARR app. Survey questions (Q1–Q10) were as follows: (1) I was comfortable using the ARR device, (2) I felt that the live team leader was guiding the code, (3) Automated countdown during PPV and chest compressions was useful, (4) Real-time APGAR score documentation was easy, (5) Automated target saturation, ETT size and Epi dose display was useful, (6) Voice prompts were helpful, (7) ARR will be helpful in the setting of limited team personnel, (8) ARR will be helpful in the setting of limited NRP experience, (9) ARR made code documentation easier, (10) I want to use the ARR system in a real resuscitation.

Outcome (3), provider comfort level, was assessed with a 10-question post-resuscitation survey that was completed after each mock code scenario. The survey was based on a five-point Likert scale asking participants to provide feedback about the ARR system. For reference, each question in the survey is detailed in the description of Figure 1. Participants were also asked to provide open-ended, written feedback on the last question of the post-resuscitation survey. To assess this survey for internal consistency or reliability, we calculated Cronbach’s α. Cronbach’s α is quantified between 0 and 1, with higher values indicating that responses for each participant across the set of survey questions are consistent and that questions likely measure the same characteristic. Cronbach’s α is calculated by the following equation:

$$\alpha =\frac{p}{p-1}\;\left(1-\frac{\sum_{i=1}^{p}s_{i}^{2}}{s_{t}^{2}}\right)$$

where *p* is the number of questions, $s_{t}^{2}$ is the variance of all the observed data and $s_{i}^{2}$ is the variance of the data from the *i*th question.

Outcomes (1) and (2) were analyzed with summary statistics showing mean or median and variability within assessment groups. Outcome measure (3) was evaluated in two different ways. First, we examined the ordinal data from the ten Likert scale questions to assess comfort level and perceived benefit of use of the ARR by associating the ratings strongly disagree, disagree, neutral, agree and strongly agree with numbers 1, 2, 3, 4 and 5, respectively. Each of the ten code teams was made up of five people who each answered ten questions, giving 500 possible response values overall for this analysis. Missing values (*n* = 31) were considered neutral in their response. Second, we used text mining analysis for the open-ended feedback question. Specifically, the words were labeled to categorize them as conveying either positive or negative sentiment using the Bing sentiment lexicon. The most common positive and negative words in the subject responses were placed in a segmented word cloud for further analysis.

## 3. Results

A total of 50 participants were recruited to attain our goal of 10 simulation mock code events using the ARR app. Basic demographics are listed in Table 1. Summary statistics by assessment group (NRP adherence, RDP-scored accuracy) for outcomes (1) and (2) were computed and are shown in Table 2, including measures of location and central tendency (mean and median) and measures of variability (standard deviation, or sd, and median absolute deviation from the median, or mad). We also show the minimum (min) and maximum (max) values. For the NRP adherence assessment, the median was 68% (range 60–76%). For the RDP-scored accuracy assessment, the median was 77.5% (range 55–90%). The median scores for NRP and RDP tools are consistent with passing scores (>60%).

Ordinal ratings for the Likert scale questions of outcome (3) assessing provider comfort with ARR use were reviewed; 47% of the responses were “agree” (237/500) and 46% of responses were “strongly agree” (180/500), demonstrating positive feedback for ARR app use, with only 0.6% (3/500) of respondents answering “strongly disagree” (Figure 1). Cronbach’s α, a measure of internal consistency and reliability, was 0.802, indicating good internal consistency, i.e., answers to the questions for each participant across the set of ten questions were similar.

**Figure 2:**
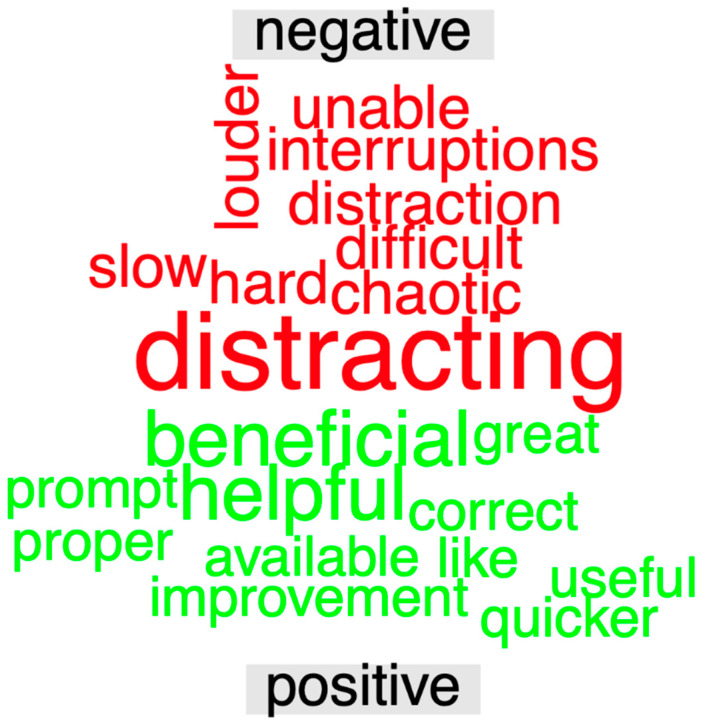
Open-ended survey question word cloud. For text mining, the words were systematically labeled to categorize words as conveying either positive or negative sentiment. The most common positive and negative words in the subject responses are shown here in a segmented word cloud (excluding words without positive or negative sentiment). The words “beneficial” and “helpful” were the most common words conveying positive sentiment, while the word “distracting” was the most common word conveying negative sentiment. Caution is needed in interpreting these results in the absence of context and how the word is used.

Open-ended responses for outcome (3) were used to generate a segmented word cloud, shown in Figure 2. Altogether, 22 subjects contributed 230 words (mean = 10.45 words, sd = 6.69 words), of which 121 were stop words. The words “beneficial” and “helpful” were the most common words conveying positive sentiment, while the word “distracting” was the most common word conveying negative sentiment.

Throughout the study, we ran into issues with information technology (IT) for our application. Prior to the initiation of our simulations, there were several “bugs” that required fixing, which delayed the initiation of data collection. Through the running of the simulations, we also discovered a few aspects of the application that could be improved based on user feedback.

## 4. Discussion

This study demonstrates the feasibility of the use of the automated resuscitation recorder in simulated neonatal resuscitation events. The median scores from the NRP and RDP tools are consistent with passing scores (>60%). Post-resuscitation survey data show that providers felt comfortable with ARR use, while highlighting areas for improvement. In the analysis of user comfort, we found that overall, most people felt comfortable with ARR use, demonstrated by a majority of positive ordinal ratings on the post-resuscitation survey, as shown in Figure 1. When answering “I want to use the ARR system in a real resuscitation”, 82% of providers responded either “agree” or “strongly agree”. Although our study was undertaken solely to determine feasibility, we included participant demographics to show that those with all experience levels found benefits with ARR use.

Several studies have shown that human factors play a significant role in adherence to NRP guidelines and code documentation. The use of application-based technology to address and reduce the impact of these factors is an area of interest in NRP research grants. Few studies have looked at app-based programs to improve compliance with NRP guidelines during delivery room resuscitation and have shown improved performance of NRP [12,13,14,15]. This is the first study to our knowledge with the goal of improving code documentation in addition to NRP adherence.

When looking at ordinal rating data for outcome (3), the question with the highest percentage of negative responses (30%) was “voice prompts were helpful,” followed by a 14% negative response to “automated countdown during PPV and chest compressions was useful.” In addition, the most common negative word used in the open-ended question answer was “distracting”, which was mostly used in reference to voice guidance for NRP interventions such as positive pressure ventilation and chest compressions. The app provided continuous counting for these interventions, which users found to be excessive. This is one area that we will explore as we make upgrades to our app prior to further study. However, we have also considered that this may be part of the learning curve for using this app, as all users only used the app one time.

Strengths of our study included clearly described procedures, a sole observer and rater to reduce rater bias and standardized grading tools. Our study had several limitations. First, it was a small feasibility study and thus we used a small convenience sample conducted in a single NICU without a control group. Second, simulation-based research studies focus highly on pattern recognition and carry with them several inherent cognitive biases, including decision making (premature closure) bias and confirmation bias [16], which are hard to reduce due to the nature of simulation.

Future experiments are needed to reduce simulation-based research biases, as above, and to test our long-term hypothesis that ARR use will improve adherence to NRP guidelines and code documentation. As referenced above, we did discover several technical difficulties with the application that required correction, as well as comments for further improvement through the running of scenarios. These comments could not be addressed until completion of our feasibility study and will be used to make upgrades to the application prior to further studies with this application. After modifications to the ARR app, we plan to perform a pilot study comparing ARR use to the current standard of care for neonatal resuscitation in the delivery room care of newborns.

## 5. Conclusions

This study demonstrates the feasibility of using application-based technology during neonatal resuscitation, and of the long-term goal of minimizing the effect of human factors on NRP adherence and code documentation. A pilot study comparing ARR use with the current standard of care is the next step in our research.

## 6. Patents

Provisional patent number 62/735,491, filed on 24 September 2019.

## Figures and Tables

**Table 1:** Demographics table.

Demographics	Group 1	Group 2	Group 3	Group 4	Group 5	Group 6	Group 7	Group 8	Group 9	Group 10
Age										
21–30	4	3	1	3	2	3	3	3	3	2
31–40	1	2	3	1	3	2	1	1	1	2
41–50	0	0	1	0	0	0	0	0	1	1
51–60	0	0	0	1	0	0	1	1	0	0
Gender										
Male	1	1	1	1	1	0	0	1	0	2
Female	4	4	4	4	4	5	5	4	5	3
Position										
Resident	1	1	1	1	1	1	1	1	1	1
Nurse	2	2	2	2	2	2	2	2	2	2
NNP	1	1	1	1	1	1	1	1	1	1
RT	1	1	1	1	1	1	1	1	1	1
Resuscitations/month										
0 to 2	5	3	2	2	3	3	4	4	3	3
3 to 5	0	1	3	0	1	2	0	1	0	1
6 to 8	0	1	0	1	0	0	0	0	1	0
9 to 10	0	0	0	1	1	0	1	0	1	1
>10	0	0	0	1	0	0	0	0	0	0
Years of NICU experience										
1 to 5	4	4	1	3	3	4	4	3	3	2
6 to 10	1	0	3	0	1	0	0	1	1	1
11 to 15	0	0	1	0	0	0	0	0	0	1
16 to 20	0	1	0	1	1	1	1	0	1	0
>20	0	0	0	1	0	0	0	1	0	1
Months from last NRP										
0 to 6	2	0	2	0	0	0	1	2	0	1
7 to 12	2	5	2	2	3	1	3	2	5	2
13 to 18	0	0	0	1	1	2	0	0	0	1
19 to 24	1	0	1	2	1	2	1	1	0	1

This table lists demographics for each simulation group 1–10. NNP = neonatal nurse practitioner; RT = respiratory therapist; months from last NRP = time since last NRP training completed.

**Table 2:** Summary statistics.

Assessment	n	Mean	Median	sd	mad	Min	Max
NRP	10	67.2	68	5.59	5.93	60	76
RDP	10	74.5	77.5	11.41	14.83	55	90

This table shows summary statistics by assessment for NRP adherence (NRP) and code documentation accuracy and completeness scores (RDP). Included statistics are number of assessments (n), mean, median, standard deviation (sd), median absolute deviation from the median (mad), minimum (min) and maximum (max) values.

## Data Availability

The original contributions presented in the study are included in the article/Supplementary Material; further inquiries can be directed to the corresponding author/s.

## Supplementary Materials

The following supporting information can be downloaded at: https://www.mdpi.com/article/10.3390/children11091137/s1, Figures S1–S4: Pages 1–4 of the ARR application.
