# Supplementary material for: Does the Use of an Automated Resuscitation Recorder Improve Adherence to NRP Algorithms and Code Documentation?
Source: Children (Basel). 2024 Sep 19;11(9):1137. doi: 10.3390/children11091137 (PMC11430511; doi:10.3390/children11091137)
Supplement: Supplementary file 1 [file children-11-01137-s001.zip › children-3196046-supplementary.pdf]

Supplements

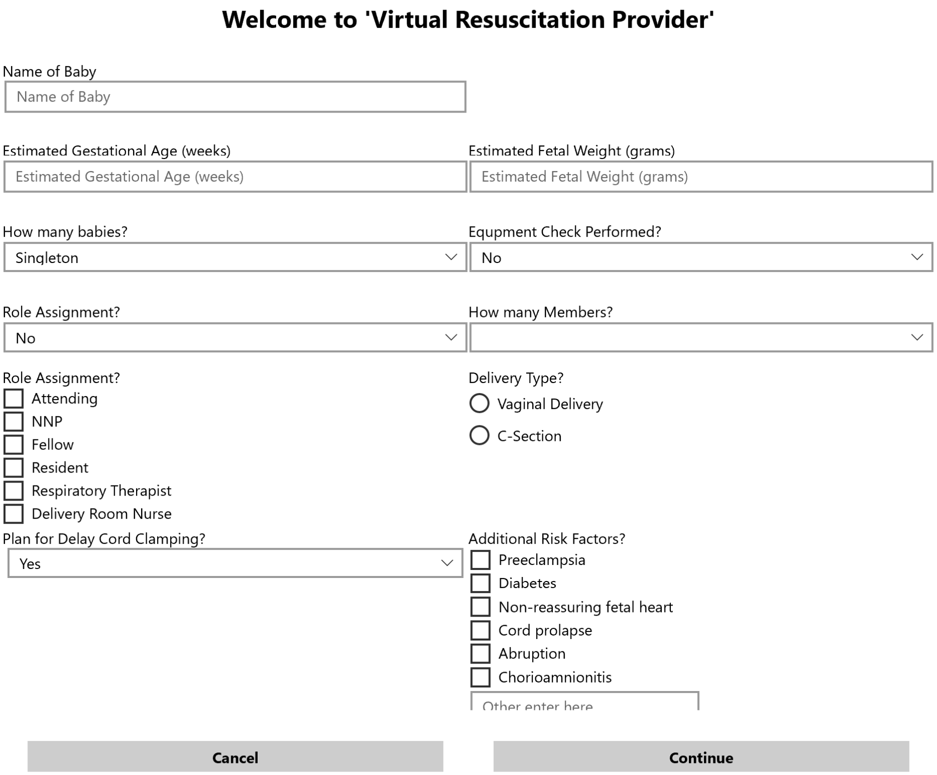

Figure S1. Page 1 of ARR application including pre-delivery information.

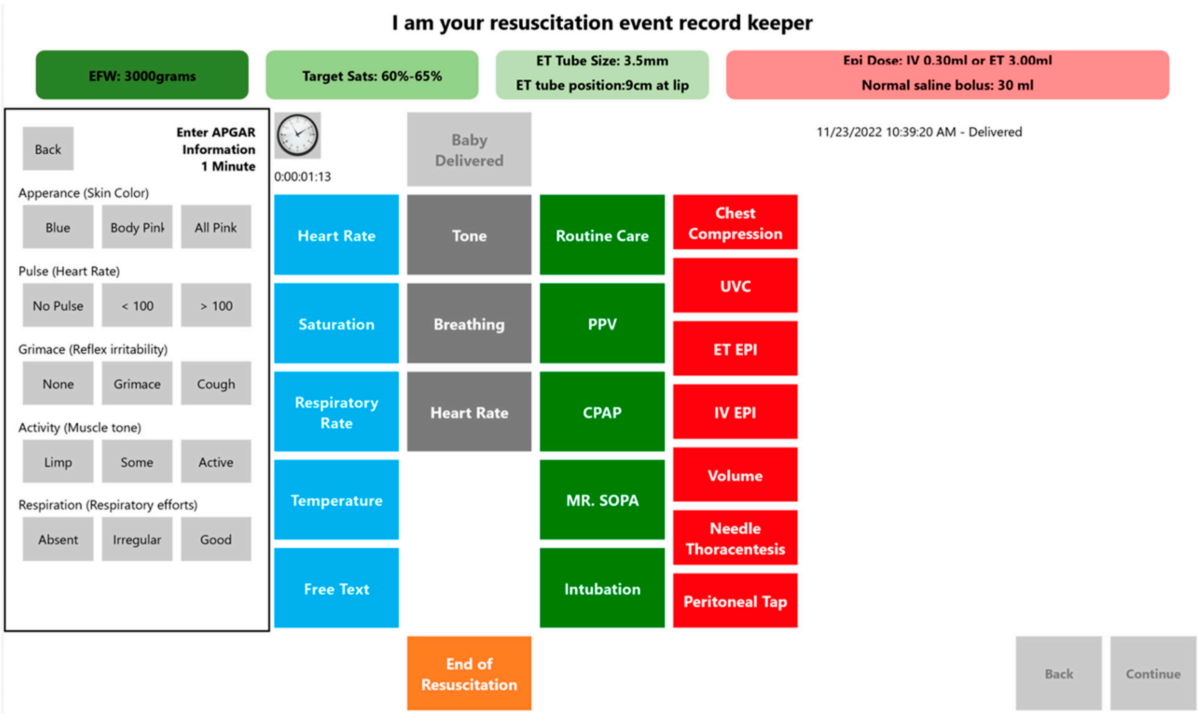

Figure S2. Page 2 of ARR application where majority of time is spent during resuscitation event to document vitals, interventions and real-time APGAR scoring. This page also provides goal saturations, medication dosing and equipment sizing. Each of the bars along row 1 cannot be edited, these will prepopulate based on input data, however, the target sats box will update with each minute of life to reflect new target sats for age. The box to the left related to APGAR scoring will remain there, at 1 and 5 MOL it will prompt the user to input the APGAR data via mouse click or touch screen. The clock next to the APGAR box, below target sats will be the timer for resuscitation, it cannot be touched. The Baby Delivered button will be pressed when baby is delivered (mouse click or touch screen) and that will start the timer. The three gray boxes below baby delivered will prompt the user to answer the first three questions for after delivery related to baby's tone/breathing/HR which can be input with mouse click or touch screen. The blue boxes to the left of these gray boxes allow you to tap (mouse click or touch screen) and then input the values with the keyboard. The green boxes also allow tap via mouse click or touch screen and if you click an intervention with a cadence (ex: PPV) it will provide a metronome for timing. MR. SOPA will provide reminders for each step, Intubation will provide a reminder for confirmation by auscultation. The red boxes to the right will provide similar options and with the chest compression box it will provide again a metronome. The space to the far right will populate every time a box is selected with the time to provide a running time log of the entire resuscitation.

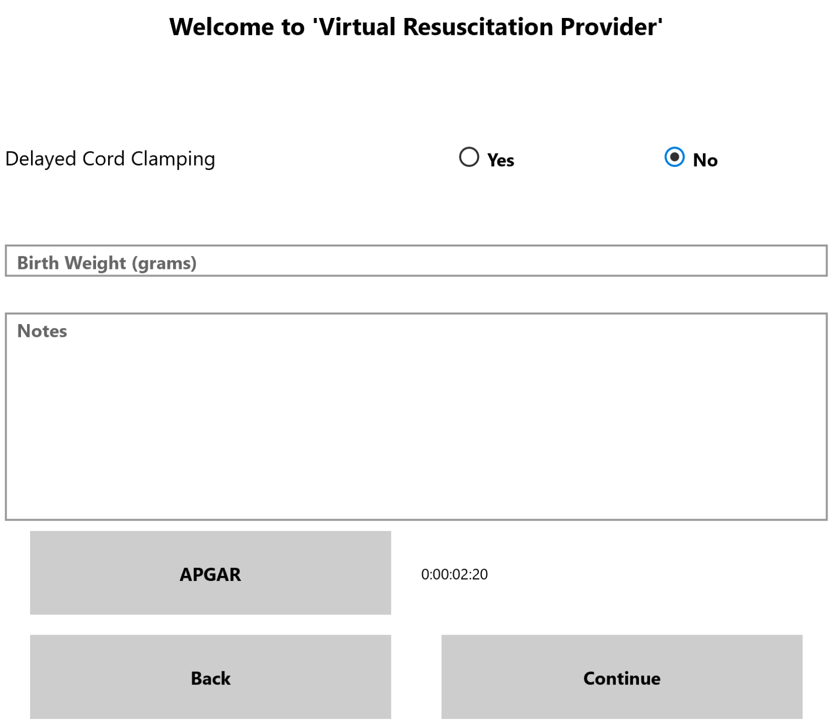

Figure S3. Page 3 of ARR application for post-resuscitation data including whether delayed cord clamping was performed, actual weight, finalized APGAR scores and allows for notes.

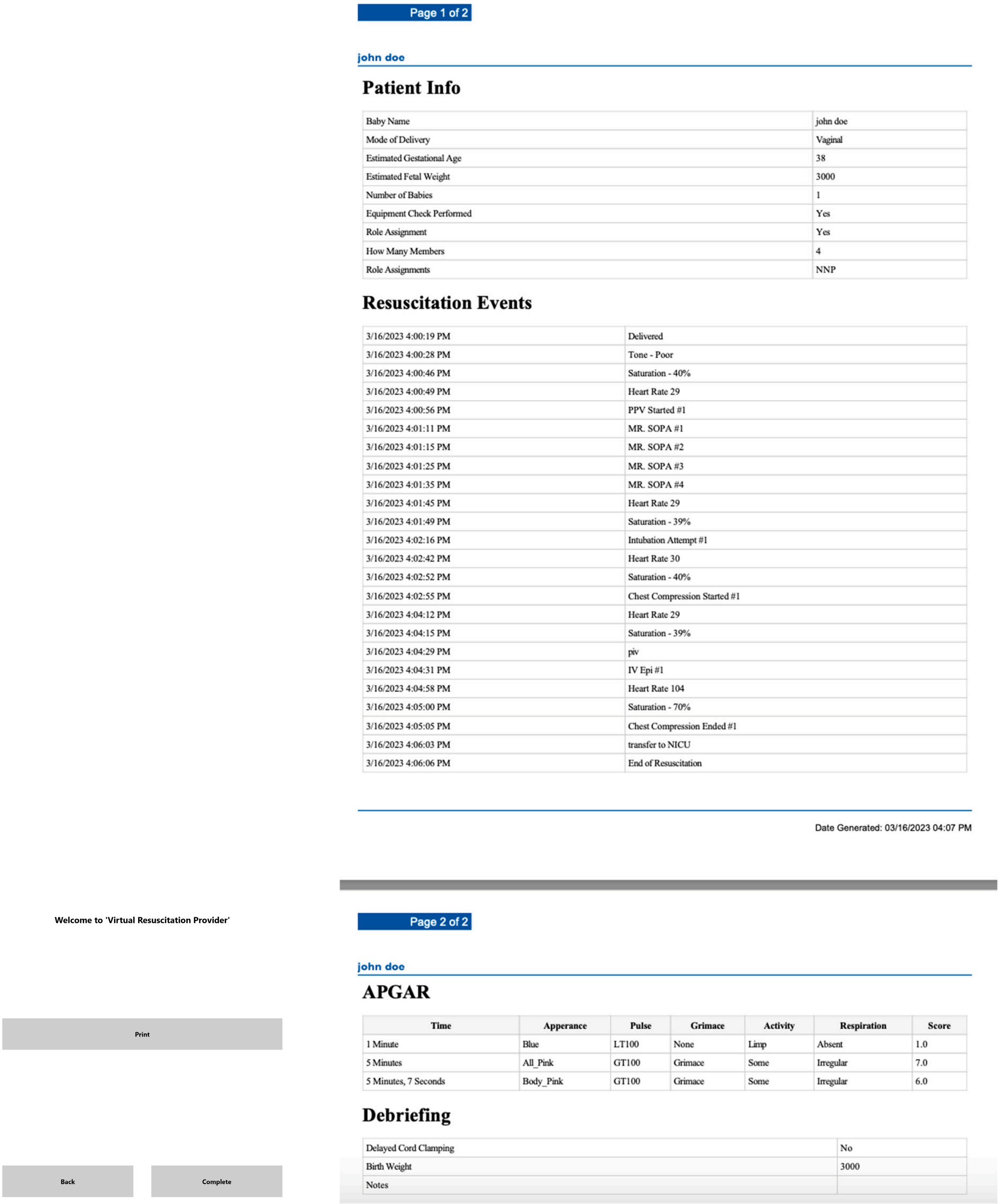

Page 1 of 2

john doe

Patient Info

Baby Name	john doe
Mode of Delivery	Vaginal
Estimated Gestational Age	38
Estimated Fetal Weight	3000
Number of Babies	1
Equipment Check Performed	Yes
Role Assignment	Yes
How Many Members	4
Role Assignments	NNP

Resuscitation Events

3/16/2023 4:00:19 PM	Delivered
3/16/2023 4:00:28 PM	Tone - Poor
3/16/2023 4:00:46 PM	Saturation - 40%
3/16/2023 4:00:49 PM	Heart Rate 29
3/16/2023 4:00:56 PM	PPV Started #1
3/16/2023 4:01:11 PM	MR. SOPA #1
3/16/2023 4:01:15 PM	MR. SOPA #2
3/16/2023 4:01:25 PM	MR. SOPA #3
3/16/2023 4:01:35 PM	MR. SOPA #4
3/16/2023 4:01:45 PM	Heart Rate 29
3/16/2023 4:01:49 PM	Saturation - 39%
3/16/2023 4:02:16 PM	Intubation Attempt #1
3/16/2023 4:02:42 PM	Heart Rate 30
3/16/2023 4:02:52 PM	Saturation - 40%
3/16/2023 4:02:55 PM	Chest Compression Started #1
3/16/2023 4:04:12 PM	Heart Rate 29
3/16/2023 4:04:15 PM	Saturation - 39%
3/16/2023 4:04:29 PM	piv
3/16/2023 4:04:31 PM	IV Epi #1
3/16/2023 4:04:58 PM	Heart Rate 104
3/16/2023 4:05:00 PM	Saturation - 70%
3/16/2023 4:05:05 PM	Chest Compression Ended #1
3/16/2023 4:06:03 PM	transfer to NICU
3/16/2023 4:06:06 PM	End of Resuscitation

Date Generated: 03/16/2023 04:07 PM

Page 2 of 2

john doe

APGAR

Time	Apperance	Pulse	Grimace	Activity	Respiration	Score
1 Minute	Blue	LT100	None	Limp	Absent	1.0
5 Minutes	All_Pink	GT100	Grimace	Some	Irregular	7.0
5 Minutes, 7 Seconds	Body_Pink	GT100	Grimace	Some	Irregular	6.0

Debriefing

Delayed Cord Clamping	No
Birth Weight	3000
Notes	

Figure S4. Page 4 of the ARR application that allows printing of the complete resuscitation with a PDF that can be used for code documentation.
